# Prevalence of Self-Reported Food Allergy in Lebanon: A Middle-Eastern Taste

**DOI:** 10.1155/2015/639796

**Published:** 2015-12-16

**Authors:** C. Irani, G. Maalouly

**Affiliations:** Hôtel-Dieu de France, Saint Joseph University, Beirut, Lebanon

## Abstract

*Background*. Food allergy (FA) is an important health problem in Western countries but there is limited data on the prevalence of food allergy in the Middle East. The main objective of our study is to assess the prevalence of self-reported food allergy in Lebanon.* Methods*. The survey was conducted by Telephone Calls questionnaire through random selection of landline phone numbers from the white pages all over Lebanon. A study questionnaire addressing the main objectives of the study was filled during the telephone call conversation.* Results*. Food allergy prevalence is estimated to be 4.1% in infants and children and 3.2% in adults. 65% of patients suffering from food allergy are females and 35% are males. Skin reactions are the most common symptoms among food allergy sufferers, reported in 86% of the cases. Signs of anaphylaxis appeared in 10.8% of cases. Fruits were the most common allergens at 35%, followed by eggs (19%) and nuts (16%). Cow's milk and spices ranked fourth (14%). Only half of food allergy sufferers sought medical advice. Allergists, dermatologists, gastroenterologists, or GPs (general practitioners) were consulted. Blood testing for specific IgE was the main diagnostic tool used by physicians.* Conclusion*. This is a pilot study of self-reported food allergy prevalence in Lebanon based on telephone survey. General prevalence is estimated to be 4.1% in infants and children and 3.2% in adults. Our study may improve awareness for proper diagnosis, food elimination, and acquisition of epinephrine autoinjectors in this part of the world.

## 1. Introduction

Food is an integral part of life; however, for some, it can cause allergies and may even lead to fatality. It is necessary to appreciate the large impact that culture and ethnicity have on food choices, eating styles, and patterns, especially in defining and accepting the diagnosis of food allergies. In 1990, food allergies were estimated to affect 1.5% of adults in the United States and 6% of children younger than 3 years of age [1, 2].

Although food allergy (FA) is an important health problem in Western countries, its prevalence varies among geographic regions. There is limited data on the prevalence of food allergy in the Middle East; the data from Turkey and Mediterranean region are even scarce [3]. In a study done in the UAE (Al-Ain city), the prevalence of FA was 8%. Eggs, fruits, and fish were the main allergens reported [4]. Without comprehensive data on the prevalence of food allergy, it will be impossible to develop appropriate local diagnostic tools and avoid the incidence of anaphylaxis to food. A clear understanding of the extent of food allergies will help public health services to be reactive rather than proactive [5]. The main objective of our study is to assess the* prevalence of self-reported food allergy* in Lebanon.

Secondary objectives include describing the principal* food* allergens, reporting the* symptoms* of food allergy in our group, the diagnostic tools, and the presence of concomitant* allergic diseases*.

## 2. Methods

The survey was conducted by a questionnaire given during Telephone Calls through random selection of landline phone numbers from the white pages all over Lebanon. The number of phone calls performed varied between 1500 and 2000 until reaching 500 answers stating Yes or No (accepting to participate in the survey). A team from Anqors Consulting handled the phone calls.

The “data collectors” hold a B.S. degree in biology, biochemistry, public health, or pharmacy with at least 6 years of experience in the pharmaceutical domain. They are well trained to run a professional conversation with patients and handle such surveys and any objection that might arise while answering the questionnaire. The sample for phone calls was randomly selected from white pages based on regional split, coded, and distributed to “data collectors” in Anqors Consulting. Codes assigned to each questionnaire were used during data verification, feeding, and analysis.

Anqors employees ran phone calls as follows:

- * Step  1*. Contact the number.
- * Step  2*. If there is an answer, check if the person answering is eligible to participate (adult, the household, etc.).
- * Step  3*. Introduce the survey to check acceptance for participation as follows: “Good morning/evening; we are calling from a professional company who holds surveys in the medical domain. We are running a survey all over Lebanon to assess the prevalence of food allergy. Are you willing to participate?” No names or addresses were requested while answering the questionnaire.
- * Step  4*. If the answer is “Yes,” proceed with the questionnaire.
- If the answer is “No,” stop there.

Later on, the phone calls were split per region depending on the number of landlines per region (relative to demographic distribution) along with the response rate per region received during the pilot phase run at the beginning of June 2014. The medical research was run through phone calls survey targeting houses and the results presented herein are qualitative and quantitative (percentage estimates). The only personal data about the responder and/or allergic person are his age and sex.

Based on previously established prevalence rate of 3% through literature review and an estimated 95% confidence interval of 1.5–4.5%, a sample size of 506 subjects was needed for the study. A pilot study with a sample of 100 subjects was taken from digitalized white pages to assess the response rate in order to decide on the final sample of phone numbers needed. Based on a response rate of 27.6%, the final sample of 1832 subjects was chosen from the digitalized white pages database. The preliminary target was 1500 call attempts, which were increased to 1832 to obtain 506 responses.

Adults or grownups were interviewed over the phone and asked if they or any of their kids suffered from food allergies. The fieldwork started at the beginning of July 2014 and continued over a period of 7 weeks. People were first asked about whether they/nonadult housemates suffer from any type of food allergy or not. Food allergy sufferers' calls elapsed for around 10 minutes.

A questionnaire addressing the main objectives of the study was filled during the telephone call conversation.

## 3. Results

**Figure 1:**
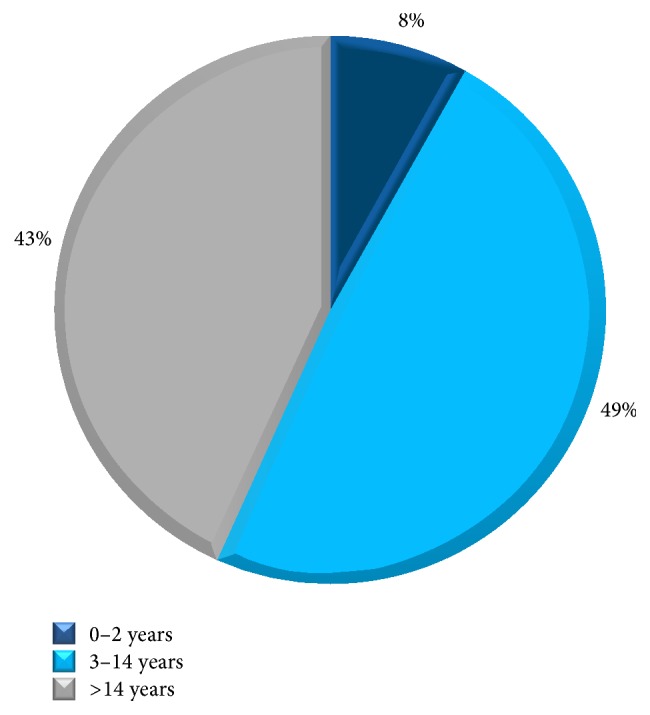
Age of onset. Family history: the prevalence of family history is estimated by 13.5%. Almost half of food allergy sufferers realize their allergy between 3 and 14 years of age. See Q3 of Food Allergy Survey in the Supplementary Material available online at http://dx.doi.org/10.1155/2015/639796.

**Figure 2:**
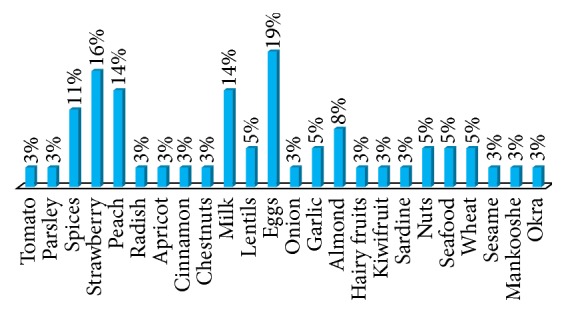
Prevalence of self-mentioned food allergens: open-ended question. The highest mentioned food allergens were eggs (19%), followed by strawberry (16%), peach, and milk (14%). See Q4 of Food Allergy Survey in the Supplementary Material.

**Figure 3:**
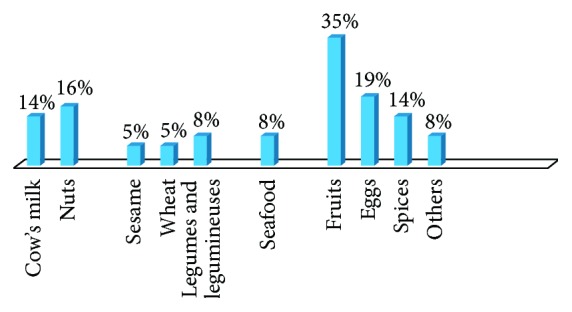
Prevalence of food allergens: assisted. Fruits, by 35%, were ranked first to cause food allergy, followed by eggs (19%) and nuts (16%). Cow's milk and spices came fourth with 14% allergy prevalence. See Q5 of Food Allergy Survey in the Supplementary Material.

Self-reported food allergy is estimated to be 4.1% in infants and children and 3.2% in adults. 65% of patients suffering from food allergy are females and 35% are males. The majority of self-reported food allergy prevalence was reported at childhood and adulthood with only 8% below 2 years of age (Figure 1). Fruits ranked first as food allergens with 35% prevalence in the open-ended question, mainly strawberry (16%) and peach (14%), followed by eggs (19%) and nuts (16%) (Figure 2). Cow's milk and spices came fourth when answering specifically about allergen classification in the questionnaire, with 14% prevalence (Figure 3). 73% of participants mentioned that they are allergic to a single food allergen and 22% mentioned that they are allergic to two food allergens, while only 5% reported allergy to three food allergens.

**Figure 4:**
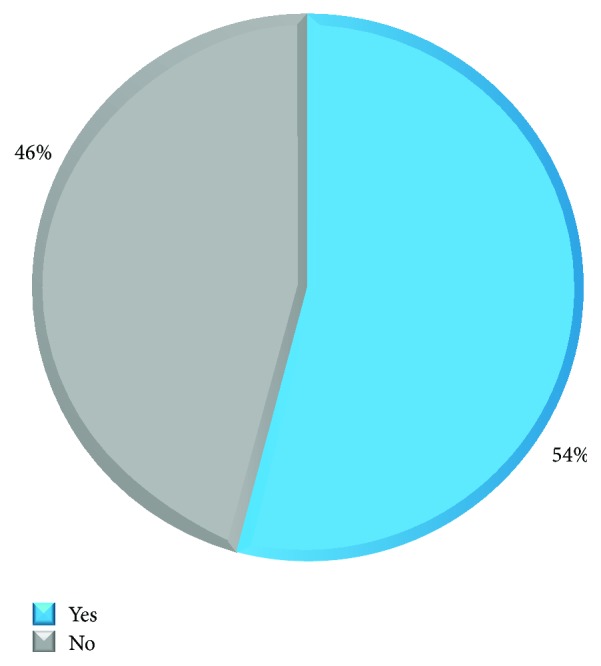
Percentage of allergic patients seeking medical advice. Only 54% of patients suffering from food allergy seek a professional medical opinion. Allergists, dermatologists, gastroenterologists, and GPs were targeted for medical advice. See Q7 of Food Allergy Survey in the Supplementary Material.

Skin reactions, including hives, itching, and redness, are the most common symptoms among food allergy sufferers, reported in 86% of the cases. 30% of food allergy sufferers reported facial swelling and 14% suffer from shortness of breath, throat swelling, angioedema, and cough, while 11% mentioned face swelling and angioedema with nausea/vomiting. Signs of anaphylaxis appeared in 10.8% of cases. Only half of food allergy sufferers sought medical advice. Allergists, dermatologists, gastroenterologists, or GPs are consulted for medical advice (Figure 4).

**Figure 5:**
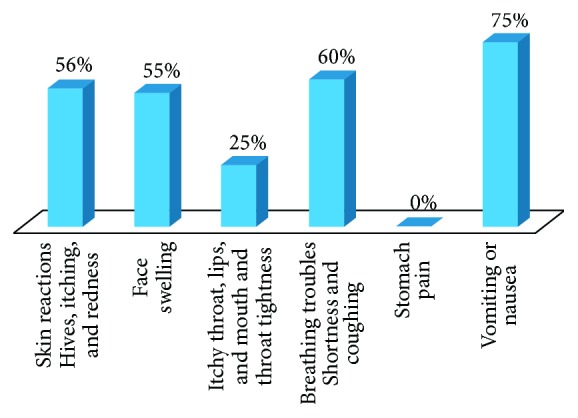
Percentage of correlation between symptoms and seeking medical advice. 75% of patients suffering from nausea and vomiting would seek medical assistance to check for their food allergy. Only 55–60% of patients suffering from skin reactions, face swelling, and breathing troubles check for medical advice for their food allergy. The least frequency in checking for medical advice is with patient suffering from throat tightness and itchy throat, lips, and mouth.

75% of patients suffering from nausea and vomiting are more likely to seek medical advice to diagnose food allergy. Only 55–60% of patients suffering from skin reactions, face swelling, and trouble in breathing seek medical advice for food allergy. Patients suffering from throat tightness and itchy throat, lips, and mouth are the least to consult a physician (Figure 5).

The most common diagnostic tool requested by medical professionals was blood test (specific IgE) (55%) followed by other options such as food elimination based on clinical history (25%) and allergy skin prick testing (20%). Other tools were mentioned, such as gastroscopy (5%).

73% of patients suffering from food allergy ask about food ingredients in case of non-self-prepared food. 75% of patients who seek medical advice check for food ingredients in non-self-prepared food.

51% of participants with food allergy have concomitant allergic diseases, such as allergic rhinitis in 30% of the cases, drug allergies (mainly antibiotics, aspirin, and nonsteroidal anti-inflammatories) in 16% of cases, and asthma in 5% of the cases.

## 4. Discussion

In the past 2 decades, food allergy has emerged as an important public health problem, affecting people of all ages in societies with a Western lifestyle, such as the United States, Canada, United Kingdom, Australia, and Western Europe [6, 7]. Food allergy is the most common cause of anaphylaxis in the outpatient setting for all ages, and it can lead to fatalities.

Prevalence and moreover incidence of food allergy are often difficult to assess because their evaluation is influenced by multiple factors such as allergy definition, study population, methodologies, geographic variation, age, and dietary exposure [8].

A recent meta-analysis showed that self-reported prevalence of food allergy varied from 1.2% to 17% for milk, from 0.2% to 7% for egg, from 0% to 2% for peanuts and fish, from 0% to 10% for shellfish, and from 3% to 35% for any food. Marked heterogeneity in the prevalence of food allergy was noticed and could be a result of differences in study design or methodology or differences between populations. The authors concluded that self-reported food allergy could not give a good estimate of food allergy [9]. In recent population-based studies in the United States, 1.3% of adults self-reported peanut allergy, tree nut allergy, or both; 2.8% of adults self-reported seafood allergy; and the adult population allergic to food was estimated to be 4% [6]. Limited data suggest that the prevalence of food allergy has increased in industrialized countries worldwide; for example, estimates of peanut and tree nut allergy tripled in American children between 1997 and 2008, but the reason for this increase remains unknown. Similar increases have been seen in the UK and Australia [10]. Local dietary habits often result in the variety of food allergens in the diet. Examples include sesame in the Middle East, buckwheat in Japan, and mustard and lupine in France [11, 12].

However, to our knowledge, few population-based studies in the Middle East have determined the prevalence of food allergy.

In the Middle East, studies about food allergy prevalence are scarce. A recent study done in the United Arab Emirates showed that the most common food allergens were seafood and nuts [13].

The prevalence of physician-diagnosed FA in children in the UAE was 8% (95% CI: 5.4–10.8%). Eggs, fruits, and fish were the main allergens reported [4].

In a previous study done by our group [14], we identified 386 out of 1842 (20.95%) patients with positive specific IgE to food allergens. The clinical presentations were cutaneous, digestive, and anaphylaxis. The major sensitizations were to cow's milk in infants and young children and to hazelnut and wheat flour in adults. Although specific IgE was more commonly detected to peanut in infants, children, and adults than to sesame, peanut-induced allergic reactions were mild, in contrast to sesame where anaphylaxis was the only clinical manifestation. Studies that measure sensitization to food allergens can overestimate the prevalence of true allergic reactions to foods, because not all sensitized patients will develop symptoms upon ingestion. In contrast, the rates of food allergy are significantly lower if allergy is defined by verified clinical reactivity upon ingestion. In one study of food allergy in Saudi patients, specific IgE antibodies for various food allergens were detected in 38 (17.5%) out of 217 patients. Most positive reactions were detected in urticaria patients (9.7%) followed by asthmatics (5.5%) and allergic rhinitis patients (2.3%). Reactions to peanut (22.6%), egg white (14.5), and cow's milk (12.9%) were very prominent. Prevalence of food allergy in Lebanon was never done previously. Mixing the terms food allergy and food intolerance is very common. Pertinent questions along with the timing and type of manifestations are essential to differentiate the two conditions. Proper testing should follow. Our questionnaire addressed this issue by excluding all cases that reported symptoms more than 2 hours after food ingestion and patients presenting with irritable bowel symptoms such as bloating.

Cultural barriers remain a major cause of denial of food allergy especially in children. Parents and grandparents are not easily convinced to avoid certain food in their child's diet. Education is imperatively needed in order to avoid delay in diagnosis and proper management with an action plan. Unfortunately, epinephrine autoinjectors are not available in the country, and when prescribed they are bought from other countries.

Plant food allergy is more common in patients with pollinosis than in the general population [15]. This may explain the highest incidence of allergy to fruits in our population which is similar to the population described in the Mediterranean area [16].

## 5. Conclusion

This is a pilot study of self-reported food allergy prevalence in Lebanon based on a telephone survey. Self-reported prevalence is estimated to be 4.1% in infants and children and 3.2% in adults. This prevalence is similar to other Middle Eastern countries but with some particularities. Advocacy for proper diagnosis, food elimination, and acquisition of epinephrine autoinjectors will hopefully result from our national study.

## Supplementary Material

A survey concerning food allergy in Lebanon.

## References

[1] Sampson H. A. (1999). Food allergy. Part 1: immunopathogenesis and clinical disorders. *Journal of Allergy and Clinical Immunology*.

[2] McBride D., Keil T., Grabenhenrich L. (2012). The EuroPrevall birth cohort study on food allergy: baseline characteristics of 12,000 newborns and their families from nine European countries. *Pediatric Allergy and Immunology*.

[3] Kaya A., Erkoçoğlu M., Civelek E., Çakir B., Kocabaş C. N. (2013). Prevalence of confirmed IgE-mediated food allergy among adolescents in Turkey. *Pediatric Allergy and Immunology*.

[4] Al-Hammadi S., Al-Maskari F., Bernsen R. (2010). Prevalence of food allergy among children in Al-Ain City, United Arab Emirates. *International Archives of Allergy and Immunology*.

[5] Hadley C. (2006). Food allergies on the rise? Determining the prevalence of food allergies, and how quickly it is increasing, is the first step in tackling the problem. *EMBO Reports*.

[6] Sicherer S. H., Sampson H. A. (2009). Food allergy: recent advances in pathophysiology and treatment. *Annual Review of Medicine*.

[7] Branum A. M., Lukacs S. L. (2009). Food allergy among children in the United States. *Pediatrics*.

[8] Sicherer S. H., Sampson H. A. (2014). Food allergy: epidemiology, pathogenesis, diagnosis, and treatment. *Journal of Allergy and Clinical Immunology*.

[9] Vierk K. A., Koehler K. M., Fein S. B., Street D. A. (2007). Prevalence of self-reported food allergy in American adults and use of food labels. *Journal of Allergy and Clinical Immunology*.

[10] Cecilia Berin M., Sampson H. A. (2013). Food allergy: an enigmatic epidemic. *Trends in Immunology*.

[11] Sicherer S. H. (2011). Epidemiology of food allergy. *Journal of Allergy and Clinical Immunology*.

[12] Rona R. J., Keil T., Summers C. (2007). The prevalence of food allergy: a meta-analysis. *Journal of Allergy and Clinical Immunology*.

[13] John L. J., Ahmed S., Anjum F. (2014). Prevalence of allergies among university students: a study from Ajman, United Arab Emirates. *ISRN Allergy*.

[14] Irani C., Maalouly G., Germanos M., Kazma H. (2011). Food allergy in Lebanon: is sesame seed the ‘Middle Eastern’ peanut. *World Allergy Organization Journal*.

[15] Flores E., Cervera L., Sanz M. L., Diaz-Perales A., Fernández J. (2012). Plant food allergy in patients with pollinosis from the mediterranean area. *International Archives of Allergy and Immunology*.

[16] Fernández Rivas M. (2003). Cross-reactivity between fruit and vegetables. *Allergologia et Immunopathologia*.

