# Supplementary material for: Prevalence of Self-Reported Food Allergy in Lebanon: A Middle-Eastern Taste
Source: Int Sch Res Notices. 2015 Dec 16;2015:639796. doi: 10.1155/2015/639796 (PMC4897070; doi:10.1155/2015/639796)
Supplement: Supplementary file 1 — A survey concerning food allergy in Lebanon. [file 639796.f1.doc]

Food Allergy Survey

Good morning/ Good Evening; we are running a survey concerning food allergy in Lebanon.

Are you willing to participate in our study? It will not take more than 5 minutes.

If yes, continue the survey.

If no, thank you.

Initials: -------------------- Sex: □F □M

Age: -------------------- Region:-----------------

Q1- Do you currently have, or suspect to have any food allergy? □Y □N

*If answer is Y, then continue survey. If answer is N, then go to Q2.*

Q2- Do anyone living with you at the same house suffer from any food allergy? □Y □N

*If yes, Fill in New report about this/ these allergic person(s), or take his/ their contact and ask them directly.*

Q3- At what age did you realize (or your parents realize) that you have food allergy? ---------------

□0-2 years □3-14 years □> 14 years

Q4- What kind of food are you allergic to? (open ended question)

Q5- Are you allergic to any of the below (one or more answers may apply)

□Cow’s Milk

□Nuts (□Peanuts □Tree nuts)

□Sesame

□Wheat

□Legumineuses (Chick peas , Lentils…)

□Seafood (□Fish □Shellfish)

□Fruits

□Eggs

□Spices

Q6- What Symptoms do you observe/ manifest immediately after food ingestion? (one or more answers may apply)

□Skin Reactions (Hives, Itching, Redness)

□Swelling of face

□Itchy Throat, Lips or Mouth, Throat tightness

□Trouble breathing, Shortness of breath, coughing

□Stomach pain

□Vomiting or nausea

□Diarrhea

Q7- Did you seek a professional medical Opinion to diagnose your food allergy?

□Y □N

*If Yes, then go to Q8; if No, then go directly to Q9*

Q8- How did the Doctor confirm your Food allergy?

□Skin Test

□Blood Test

□Food elimination

□History

□I don’t know

Q9- If you are eating non self- prepared food, do you usually ask about ingredients?

□Y □N

Q10- Do you suffer from any other types of allergy?

□Allergy to medications; (What type of medications-----------------------------------------------)

□Rhinitis

□Asthma

Thank you
